# Evaluation of Bone Invasion in Gingival Squamous Cell Carcinoma Using PET/CT Radiomics

**DOI:** 10.3390/diagnostics16172685

**Published:** 2026-08-22

**Authors:** Junwoo Kwark, Seunggon Jung, Min-Suk Kook, Hong-Ju Park, Junho Chang, Jaeyoung Ryu

**Affiliations:** 1 Department of Oral & Maxillofacial Surgery, Chonnam National University Hospital, Gwangju 61469, Republic of Korea; junkwark@gmail.com (J.K.); seunggon.jung@chonnam.ac.kr (S.J.); omskook@chonnam.ac.kr (M.-S.K.); omspark@chonnam.ac.kr (H.-J.P.); mr_juno@naver.com (J.C.); 2 Department of Oral & Maxillofacial Surgery, School of Dentistry, Dental Science Research Institute, Chonnam National University, Gwangju 61186, Republic of Korea

**Keywords:** gingival squamous cell carcinoma, bone invasion, ^18^F-FDG PET/CT, radiomics, metabolic tumor volume

## Abstract

**Background**: Accurate preoperative assessment of bone invasion is essential for surgical planning in gingival squamous cell carcinoma (SCC). This study evaluated the diagnostic value of conventional ^18^F-FDG PET/CT-derived imaging parameters and PET-based radiomic features for predicting histopathologic bone invasion. **Methods**: This retrospective study included 68 patients with gingival SCC who underwent preoperative ^18^F-FDG PET/CT followed by primary surgical treatment. Bone invasion was confirmed histopathologically. Conventional PET parameters (MTD, SUVmax, SUVmean, MTV, and TLG) and PET-based radiomic features were extracted from the primary tumor. Group comparisons, receiver operating characteristic (ROC) analysis, and univariate logistic regression were performed. **Results**: Patients with bone invasion showed significantly higher MTD, SUVmax, MTV, and TLG than those without bone invasion (all *p* < 0.05), whereas SUVmean was not significantly different. Entropy, correlation, and zone entropy were significantly higher in the bone invasion-positive group. MTV showed the numerically highest AUC among conventional PET parameters (AUC = 0.748), whereas correlation (AUC = 0.725) and zone entropy (AUC = 0.714) showed the highest performance among radiomic features. Univariate logistic regression showed significant associations of MTD, MTV, TLG, entropy, correlation, and zone entropy with bone invasion. **Conclusions**: Conventional PET parameters and PET-based radiomic features were significantly associated with histopathologic bone invasion in gingival SCC. Radiomic features reflecting intratumoral heterogeneity may complement conventional metabolic parameters and improve preoperative assessment of bone invasion.

## 1. Introduction

Gingival squamous cell carcinoma (SCC) frequently develops adjacent to the maxillary or mandibular alveolar bone, and accurate preoperative assessment of bone invasion is essential for surgical planning [1]. The presence of bone invasion can affect clinical staging, the decision between marginal and segmental bone resection, and the need for reconstruction. However, distinguishing true tumor invasion from inflammatory or reactive bone changes remains challenging on conventional imaging [2].

CT and MRI are commonly used to evaluate cortical destruction and bone marrow involvement. MRI is sensitive for marrow changes, whereas PET/CT may provide complementary functional information. Abd El-Hafez et al. [3] reported that PET/CT was more specific than MRI for detecting bone marrow invasion in oral cavity SCC, although MRI showed higher sensitivity. These findings suggest that metabolic imaging may contribute to preoperative evaluation of bone involvement.

^18^F-FDG PET/CT is widely used in oral SCC for staging and assessment of tumor metabolism. Conventional imaging parameters such as SUVmax, MTV, and TLG have been associated with aggressive clinicopathologic features, including bone invasion and prognosis. Shimizu et al. reported that TLG was a useful predictive factor for vertical mandibular invasion in lower gingival SCC [4]. Nevertheless, these conventional imaging parameters primarily reflect tumor size or metabolic burden and may not fully capture intratumoral heterogeneity [5].

Radiomics allows quantitative extraction of imaging features that reflect tumor morphology, intensity distribution, and spatial heterogeneity. Gillies et al. [6] described radiomics as an approach that converts medical images into mineable quantitative data. In OSCC and lower gingival SCC, PET-based radiomic features have recently been applied to evaluate bone marrow invasion, prognosis, and cervical lymph node metastasis. However, the value of selected interpretable PET-based radiomic features for predicting bone invasion in gingival SCC has not been fully established [7,8,9].

Therefore, this study aimed to evaluate conventional PET/CT-derived imaging parameters and PET-based radiomic features for predicting bone invasion in patients with gingival SCC. We further investigated whether radiomic features reflecting intratumoral heterogeneity could complement conventional imaging parameters in the preoperative assessment of bone invasion.

## 2. Materials and Methods

### 2.1. Study Design and Patient Selection

This retrospective study was approved by the Institutional Review Board of Chonnam National University Hospital on 2 June 2026 (IRB No. CNUH-2026-199). Patients diagnosed with oral squamous cell carcinoma who underwent primary surgical treatment at the Department of Oral and Maxillofacial Surgery, Chonnam National University Hospital, between January 2010 and December 2025 were retrospectively screened for eligibility.

**Figure 1:**
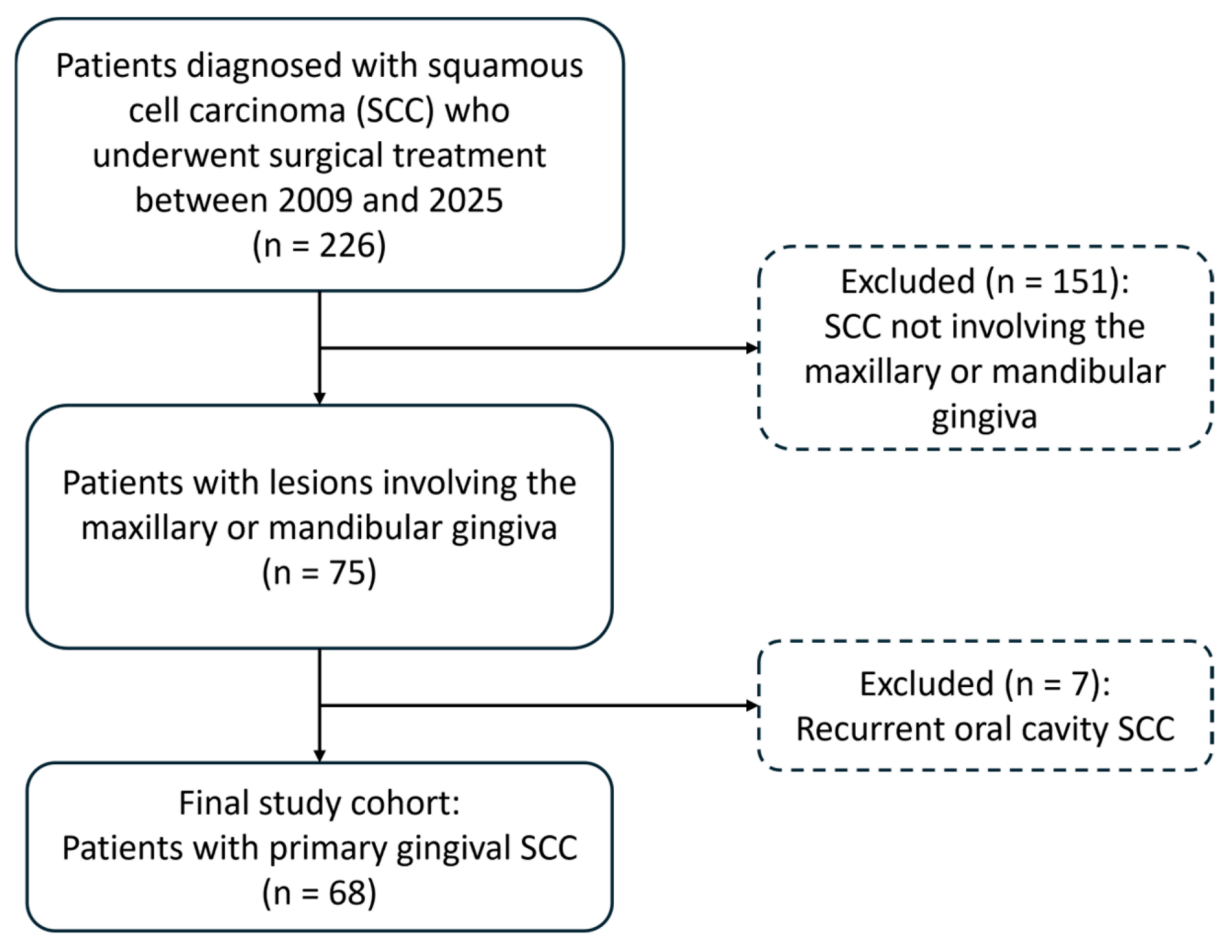
Flow diagram of the patient selection process for the study cohort.

A total of 226 patients were initially identified. Of these, 151 patients were excluded because their tumors did not involve the maxillary or mandibular gingiva. Among the remaining 75 patients with tumors involving the maxillary or mandibular gingiva, 7 patients with recurrent oral cavity SCC were excluded because the present study was restricted to primary tumors. Consequently, 68 patients with primary gingival SCC of the oral cavity were included in the final analysis. The patient-selection process is summarized in Figure 1.

Because of the retrospective study design, no prior sample-size calculation was performed. All consecutive eligible patients meeting the inclusion criteria during the study period were included in the analysis.

Eligible patients were required to have undergone preoperative torso ^18^F-fluorodeoxyglucose (^18^F-FDG) positron emission tomography/computed tomography (PET/CT) and to have available postoperative histopathologic results. All patients underwent diagnostic biopsy confirming SCC before PET/CT acquisition, in accordance with the institutional clinical and reimbursement workflow. No patients were excluded solely based on subjective PET/CT image-quality assessment.

The presence or absence of bone invasion was determined based on the final postoperative histopathologic examination of the resected specimen. Patients were categorized into bone invasion-positive and bone invasion-negative groups accordingly.

Baseline demographic and clinicopathologic variables included age, sex, anatomical location (maxilla or mandible), clinical T stage, histological differentiation, pathological nodal status, the interval between PET/CT acquisition and surgery, and the interval between diagnostic biopsy and PET/CT acquisition. All patients underwent diagnostic biopsy before PET/CT acquisition. Depth of invasion, tumor thickness, and pathological tumor size were not included because these variables were not consistently documented across the retrospective cohort. Pathological T stage was not included as a comparative baseline variable because the presence and extent of bone invasion contribute directly to pathological T classification, resulting in an inherent dependency between the study outcome and the staging variable.

### 2.2. PET/CT Acquisition, Image Processing, and Parameter Measurement

All patients fasted for at least 6 h before ^18^F-FDG administration, and blood glucose levels were confirmed to be ≤180 mg/dL before tracer injection. PET/CT imaging was performed after an uptake period of approximately 50 ± 10 min. Torso PET acquisition extended from the skull vertex to the mid-thigh and was performed in three-dimensional acquisition mode. PET images were reconstructed using an ordered-subsets expectation maximization (OSEM) algorithm with a reconstructed voxel size of 3.75 × 3.75 × 3.75 mm. A standard *z*-axis filter was applied consistently, without additional smoothing filters or time-of-flight or point-spread-function reconstruction. Low-dose CT was acquired at 120 kVp and 10 mAs with a slice thickness of 3.75 mm.

Two PET/CT scanners were used during the study period. Before July 2024, examinations were performed using a Discovery ST scanner (GE HealthCare, Milwaukee, WI, USA). ^18^F-FDG was administered at a dose of 0.13 mCi/kg, and PET data were acquired for 2.5 min per bed position. Images were reconstructed using 2 iterations and 20 subsets with a 128 × 128 reconstruction matrix. From July 2024 onward, examinations were performed using an Omni Legend scanner (GE HealthCare, Milwaukee, WI, USA). ^18^F-FDG was administered at a dose of 0.07 mCi/kg, and PET data were acquired for 1.5 min per bed position. Images were reconstructed using 25 iterations without subsets with a 384 × 384 reconstruction matrix.

**Figure 2:**
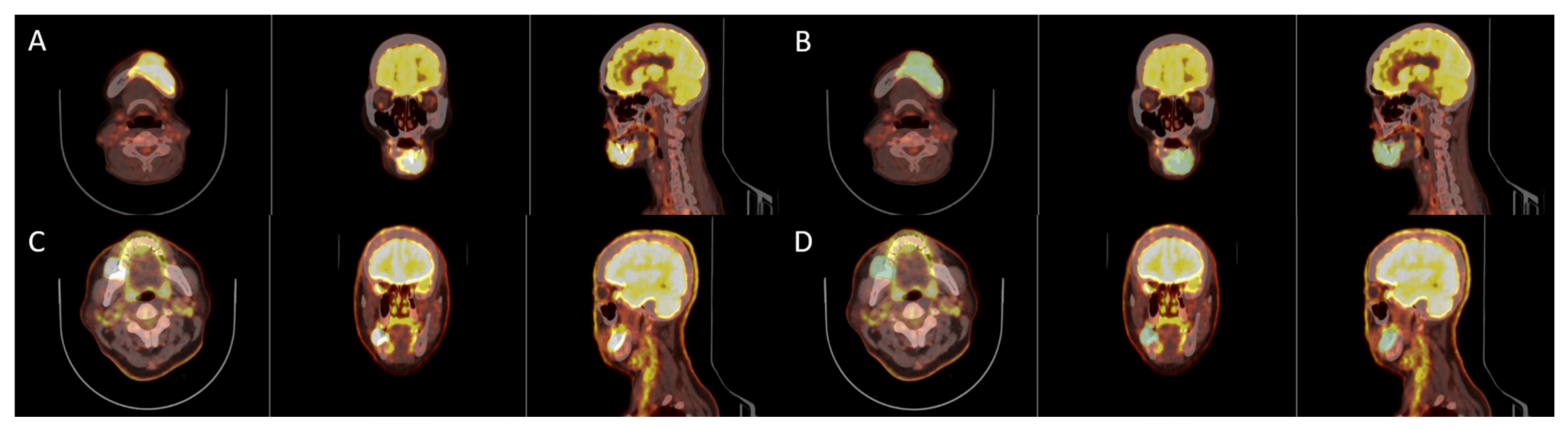
Representative ^18^F-FDG PET/CT images and corresponding primary tumor segmentations according to histopathologic bone-invasion status. (**A**) Axial, coronal, and sagittal fused PET/CT images of a representative patient with histopathologically confirmed bone invasion. (**B**) Corresponding PET-derived primary tumor segmentation displayed on the same axial, coronal, and sagittal images. (**C**) Axial, coronal, and sagittal fused PET/CT images of a representative patient without histopathologically confirmed bone invasion. (**D**) Corresponding PET-derived primary tumor segmentation displayed on the same images.

All preoperative torso PET/CT images were retrieved from the institutional picture archiving and communication system and imported into 3D Slicer software (Version 5.8.1; Slicer Community, Boston, MA, USA). Primary tumor segmentation was performed on FDG-PET images using the PETTumorSegmentation extension. This extension is based on the semiautomated “just-enough-interaction” approach described by Beichel et al. [10] which was developed and validated for hot lesion segmentation in head and neck ^18^F-FDG PET scans. The observer initialized the target lesion, after which the algorithm generated a computer-assisted tumor segmentation. The resulting volume of interest was visually inspected and manually refined when necessary to exclude physiologic FDG uptake or adjacent non-tumor structures. Representative fused PET/CT images and corresponding PET-derived primary tumor segmentations from patients with and without histopathologically confirmed bone invasion are shown in Figure 2.

Maximum tumor diameter (MTD) was calculated from the PET-derived three-dimensional tumor segmentation using a custom Python 3.9.10 script in 3D Slicer as the maximum Euclidean distance between any two points within the segmented tumor volume. Maximum standardized uptake value (SUVmax), mean standardized uptake value (SUVmean), and metabolic tumor volume (MTV) were obtained from the same segmented volume using the Segment Statistics module. Total lesion glycolysis (TLG) was calculated as SUVmean × MTV. MTD, MTV, and TLG were reported in mm, cm^3^, and g, respectively, whereas SUVmax and SUVmean were dimensionless. Among these, SUVmax, SUVmean, MTV, and TLG were considered metabolic parameters, whereas MTD was considered a tumor size parameter.

Conventional PET/CT-derived imaging parameters were measured three times at two-week intervals by a single observer, and the mean value of the three measurements was used for subsequent statistical analyses.

Radiomic features were extracted from the segmented primary tumor volumes using the radiomics module in 3D Slicer. Feature extraction was performed from the original FDG-PET images without additional image resampling within the radiomics module; therefore, the reconstructed PET voxel size of 3.75 × 3.75 × 3.75 mm was retained. No Laplacian-of-Gaussian (LoG) or wavelet filtering was applied. Intensity discretization was performed using a fixed bin width of 0.50, and symmetrical gray-level co-occurrence matrices (GLCMs) were enforced. The enabled feature classes included first-order statistics, shape features, GLCM, gray-level run-length matrix (GLRLM), and gray-level size zone matrix (GLSZM) features. Gray-level dependence matrix (GLDM), neighboring gray-tone difference matrix (NGTDM), and two-dimensional shape features were not included.

From these feature classes, a limited set of representative radiomic features was selected a priori to characterize tumor morphology, intensity distribution, and intratumoral heterogeneity. The selected features were sphericity as a shape-based feature; entropy and skewness as first-order features; contrast and correlation as GLCM features; and zone entropy as a GLSZM feature. This limited feature-selection strategy was based on previous FDG-PET radiomics studies in OSCC and lower gingival SCC and was intended to reduce model complexity and improve interpretability given the limited sample size.

### 2.3. Reproducibility Analysis

To assess intra-observer reproducibility, intraclass correlation coefficients (ICC) were calculated using a two-way mixed-effects model with absolute agreement. Repeated measures analysis of variance was additionally performed to evaluate consistency across the three measurements. Repeat segmentation for radiomic features was not performed; therefore, intra- and inter-observer reproducibility of the radiomic features could not be assessed. Accordingly, the radiomic analyses were considered exploratory and the findings were interpreted with caution.

### 2.4. Statistical Analysis

Statistical analyses were performed using IBM SPSS Statistics 29.0 (IBM Corp., Armonk, NY, USA) and R version 4.6.1 (R Foundation for Statistical Computing, Vienna, Austria). Continuous variables were assessed for normality using the Shapiro–Wilk test and compared using the independent-samples *t*-test or Mann–Whitney U test, as appropriate.

Baseline demographic and clinicopathologic characteristics were compared between patients with and without bone invasion. Age was compared using the independent *t*-test. The PET/CT-acquisition-to-surgery interval and diagnostic-biopsy-to-PET/CT-acquisition interval were compared using the Mann–Whitney U test.

Categorical variables were compared using the chi-square test or Fisher’s exact test. Univariate logistic regression analyses were performed to evaluate the associations of PET-derived and radiomic features with bone invasion, and odds ratios (ORs) with 95% confidence intervals (CIs) were reported. Linearity in the logit for continuous predictors was assessed using the Box–Tidwell procedure by including an interaction term between each predictor and its natural logarithm. To facilitate comparison of effect sizes across predictors measured on different scales, all continuous PET-derived and radiomic variables were standardized to z-scores, and odds ratios were reported per one-standard-deviation increase.

Receiver operating characteristic (ROC) curve analyses were performed in R using the pROC package (version 1.19.0.1). Areas under the ROC curve (AUCs) and their 95% CIs were estimated using DeLong’s method. The ROC direction was predefined such that higher parameter values indicated a greater probability of bone invasion. Optimal cutoff values were determined using the Youden index, with corresponding sensitivity and specificity. Pairwise comparisons of selected high-performing AUCs were performed using DeLong’s test for correlated ROC curves, including MTV versus TLG, MTV versus correlation, and correlation versus zone entropy.

To explore whether diagnostic performance differed according to tumor location, cutoff values derived from the overall cohort were applied separately to maxillary and mandibular tumors. Sensitivity and specificity were calculated for each subgroup, and differences between the two anatomical sites were assessed using two-sided Fisher’s exact tests because of the limited subgroup sample sizes.

All tests were two-sided, and *p*-values < 0.05 were considered statistically significant.

## 3. Results

### 3.1. Patient Characteristics

A total of 68 patients were included in the final analysis, of whom 41 had no bone invasion and 27 had bone invasion. The baseline demographic and clinicopathologic characteristics according to bone-invasion status are summarized in Table 1. There were no statistically significant differences between the two groups in age, sex, maxillary versus mandibular location, histological differentiation, or pathological nodal status (all *p* > 0.05). In contrast, the distribution of clinical T stage differed significantly between the groups (*p* < 0.001), with cT4 being more frequent in patients with bone invasion.

The median PET/CT-acquisition-to-surgery interval was 10.0 days (IQR, 7.8–14.2; range, 2–93 days), and the median diagnostic-biopsy-to-PET/CT-acquisition interval was 11.0 days (IQR, 7.0–15.3; range, 1–312 days). Neither interval differed significantly between the groups (*p* = 0.285 and *p* = 0.975, respectively).

### 3.2. Intra-Observer Reproducibility

All PET-derived parameters demonstrated excellent intra-observer reproducibility. The average-measure intraclass correlation coefficient (ICC) values were 0.975 for maximum tumor diameter (MTD), 1.000 for SUVmax, 0.991 for SUVmean, 0.988 for metabolic tumor volume (MTV), and 0.995 for total lesion glycolysis (TLG) (Table 2).

Mauchly’s test of sphericity indicated violations of the sphericity assumption for MTD, SUVmax, MTV, and TLG, whereas the assumption was satisfied for SUVmean. Accordingly, Greenhouse–Geisser-corrected *p*-values were used for variables violating sphericity. Repeated-measures ANOVA showed no significant differences across the three measurements for any parameter (all *p* > 0.05).

### 3.3. Comparison of Conventional PET/CT-Derived Imaging Parameters According to Bone Invasion

Normality testing showed that MTD followed a normal distribution, whereas SUVmax, SUVmean, MTV, and TLG did not. Accordingly, MTD was analyzed using an independent *t*-test, while the remaining parameters were evaluated using the Mann–Whitney U test.

MTD was significantly higher in the bone invasion group than in the non-bone invasion group (*p =* 0.010). SUVmax, MTV, and TLG were also significantly elevated in patients with bone invasion (*p =* 0.028, *p =* 0.001, and *p =* 0.002, respectively). In contrast, SUVmean did not show a statistically significant difference between the two groups (*p =* 0.128) (Table 3).

### 3.4. Comparison of PET-Based Radiomic Features According to Bone Invasion

Among the selected radiomic features, entropy, correlation, and zone entropy were significantly higher in the bone invasion-positive group than in the bone invasion-negative group. Entropy was significantly increased in tumors with bone invasion compared with those without bone invasion (3.972 ± 0.651 vs. 3.545 ± 0.796, *p =* 0.023). Correlation was also significantly higher in the bone invasion-positive group (0.433 [0.319–0.505] vs. 0.264 [0.099–0.344], *p* = 0.002), as was zone entropy (5.435 ± 0.719 vs. 4.824 ± 0.983, *p* = 0.007). In contrast, sphericity, skewness, and contrast did not differ significantly between the two groups. In contrast, sphericity, skewness, and contrast did not differ significantly between the two groups (Table 4).

**Table 4:** Comparison of radiomic features between patients with and without bone invasion.

Radiomic Feature	No Bone Invasion (n = 41)	Bone Invasion (n = 27)	*p*-Value
Sphericity	0.786 (0.764–0.812)	0.790 (0.778–0.826)	0.241
Entropy	3.545 ± 0.796	3.972 ± 0.651	0.023
Skewness	0.989 ± 0.334	0.888 ± 0.324	0.219
Contrast	18.670 (11.275–45.945)	36.124 (14.946–71.121)	0.128
Correlation	0.264 (0.099–0.344)	0.433 (0.319–0.505)	0.002
ZoneEntropy	4.824 ± 0.983	5.435 ± 0.719	0.007

Independent *t*-test was used for Entropy, Skewness, and ZoneEntropy, whereas the Mann–Whitney U test was applied for Sphericity, Contrast, and Correlation. Entropy, Correlation, and ZoneEntropy showed statistically significant differences between groups (*p* < 0.05). Values are mean ± standard deviation for independent *t*-test, and median (interquartile range) for Mann–Whitney U test.

### 3.5. Diagnostic Performance for Predicting Bone Invasion

Receiver operating characteristic (ROC) curve analysis showed that MTV had the numerically highest AUC for predicting bone invasion. The area under the curve (AUC) values were 0.681 for MTD (95% CI, 0.550–0.813), 0.657 for SUVmax (95% CI, 0.522–0.791), 0.612 for SUVmean (95% CI, 0.471–0.752), 0.748 for MTV (95% CI, 0.622–0.874), and 0.724 for TLG (95% CI, 0.598–0.851) (Table 5). Optimal cutoff values determined using the Youden index were 36.08 for MTD, 10.85 for SUVmax, 5.71 for SUVmean, 11.48 for MTV, and 55.42 for TLG (Table 5).

Among the radiomic features, correlation and zone entropy showed the numerically highest AUCs. The AUC values were 0.725 for correlation and 0.714 for zone entropy. The optimal cutoff value for correlation was 0.32, yielding a sensitivity of 78% and specificity of 71%. Zone entropy showed an optimal cutoff value of 5.55, with a sensitivity of 59% and specificity of 81%. Entropy showed moderate diagnostic performance, with an AUC of 0.673 (Table 5).

Pairwise comparison of selected high-performing parameters using DeLong’s test showed no statistically significant differences in AUC between MTV and TLG (0.748 vs. 0.724; *p =* 0.278), MTV and correlation (0.748 vs. 0.725; *p =* 0.610), or correlation and zone entropy (0.725 vs. 0.714; *p =* 0.757) (Table 6). The 95% confidence intervals for the corresponding AUC differences all included zero. Therefore, although MTV demonstrated the numerically highest AUC among the evaluated parameters, its diagnostic performance was not statistically superior to that of TLG or correlation.

In the exploratory subgroup analysis according to anatomical location, no statistically significant differences in sensitivity or specificity were observed between maxillary and mandibular tumors for any of the evaluated PET-derived imaging parameters or radiomic features (all *p* > 0.05; Table S2). These findings suggest that the diagnostic performance of the evaluated imaging features did not differ significantly according to jaw location in the present cohort.

### 3.6. Logistic Regression Analysis

In univariate logistic regression analyses using standardized continuous predictors, no significant violations of the linearity-in-the-logit assumption were identified. MTD (OR per 1-SD increase, 2.027; 95% CI, 1.147–3.580; *p* = 0.015), MTV (OR, 3.113; 95% CI, 1.467–6.607; *p* = 0.003), and TLG (OR, 2.522; 95% CI, 1.307–4.868; *p* = 0.006) were significantly associated with bone invasion. Among the radiomic features, entropy (OR, 1.838; 95% CI, 1.066–3.168; *p* = 0.028), correlation (OR, 2.095; 95% CI, 1.160–3.787; *p* = 0.014), and zone entropy (OR, 2.189; 95% CI, 1.183–4.048; *p* = 0.013) showed significant associations with bone invasion (Table 7).

## 4. Discussion

Bone invasion is a critical factor in gingival squamous cell carcinoma because it directly affects surgical planning, including the decision between marginal and segmental mandibulectomy. In the present study, we evaluated conventional imaging parameters and radiomic features derived from preoperative ^18^F-FDG PET/CT for predicting histopathologic bone invasion in patients with gingival SCC. Among the conventional imaging parameters, MTD, SUVmax, MTV, and TLG were significantly higher in the bone invasion-positive group, whereas SUVmean was not significantly different. Among the radiomic features, entropy, correlation, and zone entropy were significantly increased in tumors with bone invasion. These findings suggest that bone-invasive gingival SCC may be characterized not only by greater tumor size and metabolic tumor burden, but also by increased intratumoral heterogeneity on PET-based radiomic analysis. In our study, MTV showed the highest diagnostic performance among the conventional imaging parameters, while correlation and zone entropy showed the highest performance among the radiomic features.

Accurate imaging-based assessment of bone invasion remains clinically important in oral SCC. In clinical practice, multi-slice computed tomography (MSCT) and MRI remain the principal modalities for evaluating local tumor extension and osseous involvement. CT provides high spatial resolution for cortical bone destruction, whereas MRI is particularly sensitive for bone marrow and soft-tissue involvement. Abd El-Hafez et al. [3] reported that PET/CT was more specific than MRI for detecting bone marrow invasion in oral cavity SCC, although MRI showed higher sensitivity. In contrast to these primarily morphologic approaches, ^18^F-FDG PET/CT provides complementary functional information on tumor glucose metabolism and enables quantitative assessment of metabolic tumor burden and intratumoral heterogeneity. In addition, PET/CT allows whole-body staging within a single examination, providing simultaneous assessment of the primary tumor, regional nodal disease, and distant metastases. Nevertheless, FDG uptake is not tumor-specific and may be increased by inflammation or post-biopsy changes, while the spatial resolution of PET is limited for detailed assessment of cortical invasion. Furthermore, quantitative and radiomic PET features may be affected by acquisition, reconstruction, and segmentation protocols. Therefore, PET/CT radiomics should be regarded as a complementary approach rather than a replacement for MSCT or MRI in the assessment of bone invasion.

The association between conventional imaging parameters and bone invasion in the present study is broadly consistent with previous reports on FDG-PET in oral squamous cell carcinoma. Shimizu et al. [4] investigated diagnostic and predictive parameters for vertical mandibular invasion in lower gingival SCC and emphasized the importance of evaluating whether mandibular invasion extends vertically into deeper bone structures. In their study, PET-related parameters including SUVmax, SUVpeak, MTV, and TLG were evaluated, and TLG was identified as an important predictive factor for vertical mandibular invasion [4]. Similarly, in our cohort, TLG was significantly higher in the bone invasion-positive group and was significantly associated with bone invasion in univariate logistic regression. However, MTV showed the highest AUC among the conventional imaging parameters in our study, suggesting that the metabolically active tumor volume itself may be particularly relevant to bone-invasive behavior in gingival SCC.

Previous studies have also focused on SUVmax as a clinically accessible PET parameter for predicting bone invasion in OSCC. Stalder et al. [11] reported that OSCCs with bone invasion showed significantly higher SUVmax values and suggested that FDG-PET metabolic imaging may be useful for evaluating bone invasion in tumors located close to the maxilla or mandible. Lin et al. [5] further demonstrated, in a large OSCC cohort, that preoperative primary tumor SUVmax was associated with clinicopathologic features including bone invasion and prognosis. In line with these studies, SUVmax was significantly elevated in the bone invasion-positive group in our study. However, its diagnostic performance was lower than that of MTV and TLG. This difference may reflect the limitation of SUVmax as a single-voxel measurement: although SUVmax captures the highest metabolic activity within the tumor, it does not account for the spatial extent of metabolically active tumor tissue.

The clinical relevance of volume-based PET parameters has also been reported in oral cavity SCC. Ryu et al. [12] showed that preoperative MTV and TLG measured on ^18^F-FDG PET/CT had prognostic significance in patients with squamous cell carcinoma of the oral cavity. This supports the idea that volume-based metabolic parameters may reflect tumor burden more comprehensively than SUVmax alone [12]. In our study, both MTV and TLG were significantly higher in the bone invasion-positive group, and MTV showed the highest discriminative performance among the conventional imaging parameters. Therefore, our findings suggest that volumetric metabolic tumor burden may be closely related not only to prognosis, as shown in previous studies, but also to local bone-invasive potential in gingival SCC.

In addition to conventional imaging parameters, the present study evaluated selected radiomic features to quantify tumor morphology, intensity distribution, and intratumoral heterogeneity. Radiomics is based on the concept that medical images contain quantitative data beyond visual interpretation, and Gillies et al. [6] described radiomics as a process for extracting mineable high-dimensional features from medical images to support clinical decision-making. In the present study, entropy, correlation, and zone entropy were significantly higher in the bone invasion-positive group, suggesting that tumors with bone invasion may show more complex spatial patterns of FDG uptake. Entropy and zone entropy are particularly relevant because they reflect randomness and heterogeneity of gray-level distribution within the tumor volume. Correlation, which reflects the spatial relationship of gray levels, also showed strong discriminatory performance in this study.

These radiomic findings are broadly consistent with recent PET-based radiomics studies in oral squamous cell carcinoma, although the clinical endpoints and analytical strategies differ. Fukushima et al. [7] specifically evaluated bone marrow invasion in lower gingival SCC using machine learning-based PET texture analysis. In contrast, the present study included both maxillary and mandibular gingival SCC and focused on a limited set of predefined and interpretable radiomic features rather than constructing a high-dimensional machine-learning model. Song et al. [8] investigated the prognostic value of ^18^F-FDG PET radiomics and sarcopenia in OSCC, whereas Zhang et al. [9] developed PET/CT radiomics-based models for predicting cervical lymph node metastasis and prognosis. Although these studies addressed prognosis or nodal disease rather than local bone invasion, they similarly support the potential of PET-derived texture information to reflect tumor characteristics not fully captured by conventional metabolic parameters. The present study therefore differs from these previous investigations by focusing specifically on histopathologically confirmed bone invasion in gingival SCC and by emphasizing a smaller number of interpretable radiomic features, including entropy, correlation, and zone entropy.

Taken together, conventional PET-derived parameters and radiomic features may reflect different aspects of tumor phenotype. Conventional parameters such as MTD, MTV, and TLG primarily reflect tumor size and metabolic burden, whereas radiomic features characterize the spatial complexity of FDG uptake. However, the incremental diagnostic value of combining these feature categories was not formally evaluated in the present study. Further studies are needed to determine whether integrated imaging models can improve the preoperative assessment of bone invasion and assist in planning the appropriate extent of bone resection.

An exploratory subgroup analysis showed no significant differences in sensitivity or specificity between maxillary and mandibular tumors for any evaluated parameter. Although differences in maxillary and mandibular anatomy may influence local tumor spread, the present data did not demonstrate a clear site-dependent effect on diagnostic performance. Because the subgroup sample sizes were limited, larger studies are needed to confirm whether jaw-specific biological or anatomical characteristics affect PET-based assessment of bone invasion.

This study has several limitations. First, it was a retrospective, single-center study with a relatively limited sample size. In particular, only 27 bone-invasion events were observed among 68 patients, limiting the complexity and stability of regression modeling. To reduce the risk of overfitting, regression analyses were primarily restricted to univariate models. Although standardization of continuous predictors allowed the odds ratios to be expressed on a more interpretable and comparable scale, it does not eliminate potential sparse-data bias or instability related to the limited event count. Therefore, the regression estimates should be interpreted cautiously as exploratory associations rather than definitive independent effects. Larger cohorts will be required to obtain more precise estimates and to permit robust multivariable or penalized regression modeling. Second, although repeated measurements were performed for conventional imaging parameters to improve measurement reliability, intra-observer reproducibility analysis was not performed for the radiomic features. Radiomic features are potentially sensitive to segmentation variability, image preprocessing, and reconstruction parameters; therefore, the absence of radiomic ICC assessment should be considered when interpreting the radiomic results. Third, the baseline analysis demonstrated a significant difference in clinical T-stage distribution between patients with and without bone invasion. Because tumor burden and disease stage may influence both PET-derived imaging characteristics and the likelihood of bone invasion, the observed associations between radiomic features and bone invasion cannot be considered fully independent of disease extent. In particular, differences in radiomic heterogeneity may partly reflect more advanced or larger tumors rather than a bone invasion-specific imaging phenotype. Therefore, the radiomic findings of the present study should be interpreted as exploratory associations rather than independent predictive biomarkers. Fourth, only selected radiomic features were analyzed rather than a full high-dimensional radiomics model. This was intentional to improve interpretability, but it may have limited the predictive performance compared with machine learning-based radiomics models. Fifth, segmentation-based radiomic analysis can be influenced by image acquisition protocols, reconstruction parameters, voxel size, and segmentation methods. Therefore, standardization and external validation are necessary before these findings can be directly applied in clinical decision-making.

## 5. Conclusions

Conventional imaging parameters and PET-based radiomic features were associated with histopathologic bone invasion in gingival SCC. Among the conventional imaging parameters, MTV showed the highest diagnostic performance, while among the radiomic features, correlation and zone entropy showed the strongest discriminatory ability. Selected PET-based radiomic features showed associations with bone invasion; however, these findings require further validation with formal reproducibility assessment before clinical application.

## Figures and Tables

**Table 1:** Baseline demographic and clinicopathologic characteristics according to bone invasion status.

Characteristics	Total (n = 68)	No Bone Invasion (n = 41)	Bone Invasion (n = 27)	*p*-Value
Age, years (mean ± SD)	67.7 ± 10.4	68.1 ± 9.2	67.0 ± 12.2	0.670
Sex, n (%)				1.000
Male	44 (64.7)	27 (65.9)	17 (63.0)	
Female	24 (35.3)	14 (34.1)	10 (37.0)	
Jaw, n (%)				0.627
Maxilla	33 (48.5)	21 (51.2)	12 (44.4)	
Mandible	35 (51.5)	20 (48.8)	15 (55.6)	
Clinical T stage, n (%)				<0.001
cT1	4 (5.9)	3 (7.3)	1 (3.7)	
cT2	35 (51.5)	30 (73.2)	5 (18.5)	
cT3	9 (13.2)	6 (14.6)	3 (11.1)	
cT4	20 (29.4)	2 (4.9)	18 (66.7)	
Histological differentiation, n (%)				0.905
Well differentiated	43 (63.2)	26 (63.4)	17 (63.0)	
Moderately differentiated	21 (30.9)	13 (31.7)	8 (29.6)	
Poorly differentiated	4 (5.9)	2 (4.9)	2 (7.4)	
Pathological nodal status, n (%)				0.185
pN0	34 (50.0)	24 (58.5)	10 (37.0)	
pN1	12 (17.6)	6 (14.6)	6 (22.2)	
pN2	19 (27.9)	9 (22.0)	10 (37.0)	
pN3	1 (1.5)	0 (0.0)	1 (3.7)	
pNX	2 (2.9)	2 (4.9)	0 (0.0)	
PET/CT-acquisition-to-surgery interval, days (median (IQR))	10.0 (7.8–14.2)	9.0 (7.0–14.0)	12.0 (8.0–14.5)	0.285
Diagnostic-biopsy-to-PET/CT-acquisition-interval, days (median (IQR))	11.0 (7.0–15.3)	11.0 (7.0–15.0)	11.0 (7.5–15.5)	0.975

SD = standard deviation, IQR = interquartile range. Age was compared using the independent-samples *t*-test. The PET/CT-acquisition-to-surgery interval and diagnostic-biopsy-to-PET/CT-acquisition interval were compared using the Mann–Whitney U test. Categorical variables were compared using the chi-square test or Fisher’s exact test, as appropriate.

**Table 2:** Intra-observer reproducibility and measurement consistency of PET-derived parameters.

Parameter	ICC (95% CI)	RM-ANOVA *p*-Value
MTD	0.975 (0.957–0.987)	0.352
SUVmax	1.000 (0.999–1.000)	0.231
SUVmean	0.991 (0.985–0.995)	0.481
MTV	0.988 (0.979–0.993)	0.510
TLG	0.995 (0.993–0.998)	0.304

ICC = intraclass correlation coefficient, CI = confidence interval, MTD = maximum tumor diameter, MTV = metabolic tumor volume, TLG = total lesion glycolysis. ICC was calculated using a two-way mixed-effects model with absolute agreement. Mauchly’s test of sphericity was used to assess the sphericity assumption. When sphericity was violated, Greenhouse–Geisser-corrected *p*-values were reported; otherwise, sphericity-assumed *p*-values were used.

**Table 3:** Comparison of conventional imaging parameters between patients with and without bone invasion.

Parameter	No Bone Invasion (n = 41)	Bone Invasion (n = 27)	*p*-Value
MTD (mm)	33.89 ± 9.03	40.20 ± 10.40	0.010
SUVmax	10.23 (8.42–14.49)	14.99 (11.14–17.19)	0.028
SUVmean	5.04 (4.17–6.09)	5.92 (4.73–6.90)	0.128
MTV (cm^3^)	7.50 (3.99–10.74)	16.07 (8.31–19.99)	0.001
TLG (g)	40.77 (17.25–56.41)	94.94 (52.11–132.68)	0.002

MTD = maximum tumor diameter, MTV = metabolic tumor volume, TLG = total lesion glycolysis. Independent *t*-test was used for MTD, and the Mann–Whitney U test was applied for SUVmax, SUVmean, MTV, and TLG. MTD, SUVmax, MTV, and TLG showed statistically significant differences between groups (*p* < 0.05). Values are mean ± standard deviation for independent *t*-test, and median (interquartile range) for Mann–Whitney U test.

**Table 5:** Diagnostic performance of PET-derived parameters for predicting bone invasion.

Parameter	AUC	95% CI	Cutoff	Sensitivity	Specificity
Conventional imaging parameter					
MTD (mm)	0.681	0.550–0.813	36.08	0.74	0.61
SUVmax	0.657	0.522–0.791	10.85	0.78	0.54
SUVmean	0.612	0.471–0.752	5.71	0.63	0.68
MTV (cm^3^)	0.748	0.622–0.874	11.48	0.67	0.85
TLG (g)	0.724	0.598–0.851	55.42	0.70	0.73
Radiomic feature					
Sphericity	0.584	0.444–0.724	0.78	0.78	0.42
Entropy	0.673	0.542–0.804	3.88	0.63	0.68
Skewness	0.400	0.261–0.539	0.54	0.89	0.15
Contrast	0.610	0.472–0.748	13.64	0.78	0.44
Correlation	0.725	0.594–0.856	0.32	0.78	0.71
ZoneEntropy	0.714	0.587–0.840	5.55	0.59	0.81

AUC = area under the curve; CI = confidence interval; MTD = maximum tumor diameter; MTV = metabolic tumor volume; TLG = total lesion glycolysis. Cutoff values were determined using the Youden index.

**Table 6:** Pairwise comparison of selected high-performing parameters using DeLong’s test.

Parameter	AUC 1	AUC 2	Difference in AUC	95% CI	*p*-Value
MTV vs. TLG	0.748	0.724	0.024	−0.019 to 0.066	0.278
Correlation vs. Zone entropy	0.725	0.714	0.011	−0.063 to 0.086	0.757
MTV vs. Correlation	0.748	0.725	0.023	−0.064 to 0.109	0.610

AUC, area under the receiver operating characteristic curve; CI, confidence interval; MTV, metabolic tumor volume; TLG, total lesion glycolysis. Differences in AUC were calculated as AUC of the first parameter minus AUC of the second parameter. Pairwise comparisons were performed using DeLong’s test for correlated ROC curves.

**Table 7:** Univariate logistic regression analysis for predicting bone invasion.

Parameter	OR per 1-SD Increase	95% CI	*p*-Value
Conventional imaging parameter			
MTD	2.027	1.147–3.580	0.015
SUVmax	1.415	0.851–2.352	0.181
SUVmean	1.184	0.726–1.931	0.499
MTV	3.113	1.467–6.607	0.003
TLG	2.522	1.307–4.868	0.006
Radiomic feature			
Sphericity	1.288	0.767–2.164	0.339
Entropy	1.838	1.066–3.168	0.028
Skewness	0.728	0.439–1.206	0.218
Contrast	1.159	0.712–1.886	0.552
Correlation	2.095	1.160–3.787	0.014
ZoneEntropy	2.189	1.183–4.048	0.013

OR = odds ratio; SD = standard deviation; CI = confidence interval; MTD = maximum tumor diameter; MTV = metabolic tumor volume; TLG = total lesion glycolysis. ORs are expressed per one-standard-deviation increase in each continuous predictor.

## Data Availability

The data presented in this study are available on request from the corresponding author. The data are not publicly available due to privacy and ethical restrictions.

## Supplementary Materials

The following supporting information can be downloaded at: https://www.mdpi.com/article/10.3390/diagnostics16172685/s1, Table S1: Assessment of the linearity-in-the-logit assumption for continuous parameters using the Box–Tidwell procedure; Table S2: Comparison of diagnostic sensitivity and specificity between maxillary and mandibular gingival squamous cell carcinomas.

## Abbreviations

The following abbreviations are used in this manuscript:

SCC	Squamous cell carcinoma
ROC	Receiver operating characteristic
^18^F-FDG	^18^F-fluorodeoxyglucose
PET/CT	Positron emission tomography/computed tomography
MTD	Maximum tumor diameter
SUVmax	Maximum standardized uptake value
SUVmean	Mean standardized uptake value
MTV	Metabolic tumor volume
TLG	Total lesion glycolysis
ICC	Intraclass correlation coefficient
AUC	Area under the curve
ORs	Odds ratios
