# Supplementary material for: Evaluation of Bone Invasion in Gingival Squamous Cell Carcinoma Using PET/CT Radiomics
Source: Diagnostics (Basel). 2026 Aug 22;16(17):2685. doi: 10.3390/diagnostics16172685 (PMC13565136; doi:10.3390/diagnostics16172685)
Supplement: Supplementary file 1 [file diagnostics-16-02685-s001.zip › diagnostics-4470773-supplementary.pdf]

Supplementary tables

Table S1. Assessment of the linearity-in-the-logit assumption for continuous parameters using the Box–Tidwell procedure

Parameter	Box-Tidwell interaction *p*-value
Conventional imaging parameter	
MTD	0.521
SUVmax	0.064
SUVmean	0.174
MTV	0.334
TLG	0.404
Radiomic feature	
Sphericity	0.343
Entropy	0.275
Skewness	0.839
Contrast	0.113
Correlation	0.893
ZoneEntropy	0.409

The Box–Tidwell interaction term was calculated for each continuous parameter. A *p*-value < 0.05 was considered indicative of a potential violation of the linearity-in-the-logit assumption. No significant violations were identified.

Table S2. Comparison of diagnostic sensitivity and specificity between maxillary and mandibular gingival squamous cell carcinomas

Parameter	Maxilla sensitivity	Mandible sensitivity	*p*-value	Maxilla specificity	Mandible specificity	*p*-value
Conventional imaging parameter						
MTD (mm)	0.75	0.73	1.000	0.57	0.65	0.751
SUVmax	0.92	0.67	0.182	0.62	0.45	0.354
SUVmean	0.67	0.53	0.696	0.67	0.70	1.000
MTV (cm^3)	0.67	0.67	1.000	0.86	0.85	1.000
TLG (g)	0.58	0.80	0.398	0.71	0.75	1.000
Radiomic feature						
Sphericity	0.75	0.60	0.683	0.43	0.50	0.758
Entropy	0.58	0.67	0.706	0.71	0.65	0.744
Skewness	0.92	0.87	1.000	0.14	0.15	1.000
Contrast	0.83	0.73	0.662	0.57	0.3	0.118
Correlation	0.75	0.73	1.000	0.76	0.65	0.505
ZoneEntropy	0.58	0.60	1.000	0.81	0.80	1.000

Cutoff values derived from the overall cohort were applied to both maxillary and mandibular tumors. Differences in sensitivity and specificity between anatomical sites were assessed using two-sided Fisher's exact tests.
